# Clinical characteristics of hepatitis flares during pregnancy and postpartum in Chinese chronic hepatitis B virus carriers—a prospective cohort study of 417 cases

**DOI:** 10.3389/fimmu.2022.1031291

**Published:** 2022-10-13

**Authors:** Xiaoxiao Wang, Aixin Song, Xiao Lin, Junfeng Lu, Sujun Zheng, Lina Ma, Shan Ren, Yanhong Zheng, Xinyue Chen

**Affiliations:** First Department of Liver Disease Center, Beijing Youan Hospital, Capital Medical University, Beijing, China

**Keywords:** chronic HBV carriers, pregnant women, hepatitis flares, drug cessation, postpartum

## Abstract

**Background:**

In China, it is common for pregnant women with a high load of hepatitis B virus (HBV) to take nucleos(t)ide analogue (NA) to prevent maternal-to-child transmission of HBV. However, the impact of NA intervention on virological and biochemical parameters in pregnant and postpartum women and the safety of drug cessation remain unclear. A prospective observational cohort was established in this study to analyze the clinical characteristics of hepatitis flares in pregnant and postpartum chronic HBV carriers, with or without NA intervention.

**Methods:**

Pregnant women who were chronic HBV carriers were enrolled in this study and divided into an NA intervention group and a non-intervention group according to their preferences. Liver function, HBV DNA level, and HBV serological markers were regularly measured during pregnancy and at approximately 6 weeks, 12 weeks, 24 weeks, 36 weeks, and 48 weeks postpartum.

**Results:**

A total of 417 patients were enrolled, including 303 in the NA intervention group and 114 in the non-intervention group. The incidence rates of postpartum hepatitis flares in both groups were higher than that of during pregnancy (45.7% vs 10.9%, p < 0.001; 41.2% vs 17.7%, p < 0.001). The second trimester was the peak of the incidence of flares during pregnancy and the incidence peak of postpartum flares was about 6 weeks postpartum. A total of 98% (145/148) of postpartum flares occurred within 24 weeks postpartum. After drug cessation, the incidence rate of flares was 34.1% (44/129).

**Conclusion:**

In pregnant chronic HBV carriers, a certain proportion of hepatitis flares occurred during pregnancy and postpartum regardless of whether NA intervention was used, and the incidence of postpartum flares (44.6%) was significantly higher than that (12.8%) of during pregnancy. The flare incidence peaked at approximately 6 weeks postpartum, which may be the time period suitable for treatment. Since 98% of postpartum flares occurred within 24 weeks postpartum, the follow-up after drug cessation should be at least 24 weeks postpartum.

## 1 Introduction

In China, most patients with chronic hepatitis B virus (HBV) infection acquired the infection at a young age, mother-to-child transmission as the main transmission route, accounting for approximately 40% of all HBV infections (1). Among pregnant women with high HBV load, 8-15% of them still can transmit HBV to their infants even after their infants receive conventional immunization (2–4). Growing evidence (4–6) in recent years has confirmed that the administration of nucleos(t)ide analogue (NA) in women with high HBV load during second or third trimester can inhibit viral replication and thereby significantly increase the success rate of prevention of mother-to-child transmission. Therefore, the Chinese and international guidelines for hepatitis B (7–10) have recommended that pregnant women with high HBV load receive NA intervention during second or third trimester. NA intervention does benefit newborns significantly, but the impact of NA intervention on virological and biochemical parameters in pregnant and postpartum women, and the safety of drug cessation are still unclear. This prospective observational cohort study was to investigate the clinical characteristics of hepatitis flares in pregnant and postpartum women.

## 2 Patients and methods

### 2.1 Study population

The subjects of the study were chronic HBV carriers visited in Beijing You’an Hospital Affiliated to Capital Medical University(from November 2015 to July 2018), who were planning for pregnancy or pregnant (less than 8 weeks of gestation). The inclusion criteria were as follows: age 18-40 years, hepatitis B surface antigen (HBsAg) positivity for more than 6 months, HBeAg hepatitis B e antigen (HBeAg) positivity, HBV DNA ≥ 2 × 10^6^ IU/ml, no history of antiviral therapy, and normal alanine aminotransferase (ALT) levels in at least two tests within 2 years before pregnancy. The exclusion criteria were as follows: hepatitis A, C, D, or E infection; other chronic liver diseases; human immunodeficiency virus infection; cirrhosis or hepatocellular carcinoma; and intrahepatic cholestasis of pregnancy and a history of miscarriage.

### 2.2 Research methods

A prospective, open-cohort design was adopted for this clinical study. The patients were given the choice between joining either the non-intervention group (group A) or the NA intervention group (group B) during pregnancy. Medications used for intervention included telbivudine (LDT) and tenofovir disoproxil (TDF), which were started at 24 weeks to 28 weeks of gestation at a dose of 600 mg/day for LDT and 300 mg/day for TDF. To select LDT or TDF was based on patients’ willingnesses. All patients were informed about this study and signed informed consent forms before enrollment (approval number: JingYou KeLun Zi[2015]21).

The team members of the joint research group of the Department of Obstetrics and the Department of Hepatology followed up the patients regularly from pregnancy to 48 weeks postpartum. Patients were followed up once every 4-6 weeks during pregnancy. The first postpartum follow-up was approximately 6 weeks postpartum (6 ± 2 weeks), and afterwards the patients were followed up approximately every 12 weeks until the completion of the 48-week follow-up. HBV DNA, HBV serological markers, liver function and renal function should be tested during each follow-up. Abnormal liver function was defined as ALT > upper limit of normal (ULN, ULN was 40 U/L), including mildly abnormal liver function (ULN < ALT < 2 × ULN), hepatitis flare (ALT ≥ 2 × ULN), and hepatitis exacerbation (ALT ≥ 10 × ULN or Total bilirubin ≥ 5 × ULN, ULN was 17.1umol/L). Patients with abnormal liver function test results were re-examined within 2 weeks, and any liver injury-inducing factors, such as alcohol, fat and drugs, had to be excluded by a clinicians’ assessment.

### 2.3 Laboratory test methods

HBV DNA was detected using the Roche Cobas/Taqman Real-Time fluorescence quantitative polymerase chain reaction (PCR) system (Roche Diagnostics GmbH, Germany), and the low limit of detection was 20 IU/mL. The HBV serological markers were detected using the Roche E601 automatic chemiluminescence analyzer, with lower detection limit of 0.05 IU/ml for HBsAg and 1 COI for HBeAg. An OLMPUS-AU5400 biochemistry analyzer was used to detect biochemical parameters such as liver function parameters. HBV was genotyped with the HBV genotyping reagent kit (fluorescence PCR) (Fosun Pharma, Shanghai, China).

### 2.4 Statistical analysis

SPSS 25.0 (SPSS, USA) software was used for the statistical analysis. Continuous variables were presented as the mean ± standard deviation or median (interquartile range). Categorical variables were expressed as frequencies and percentages. Continuous variables were assessed using Student’s t test or Ranksum test. Categorical variables were compared with the Chi-squared test or Fisher’s exact test. A two-sided P value of < 0.05 indicated statistical significance.

## 3 Results

### 3.1 General information of enrolled patients and follow-up

**Figure 1:**
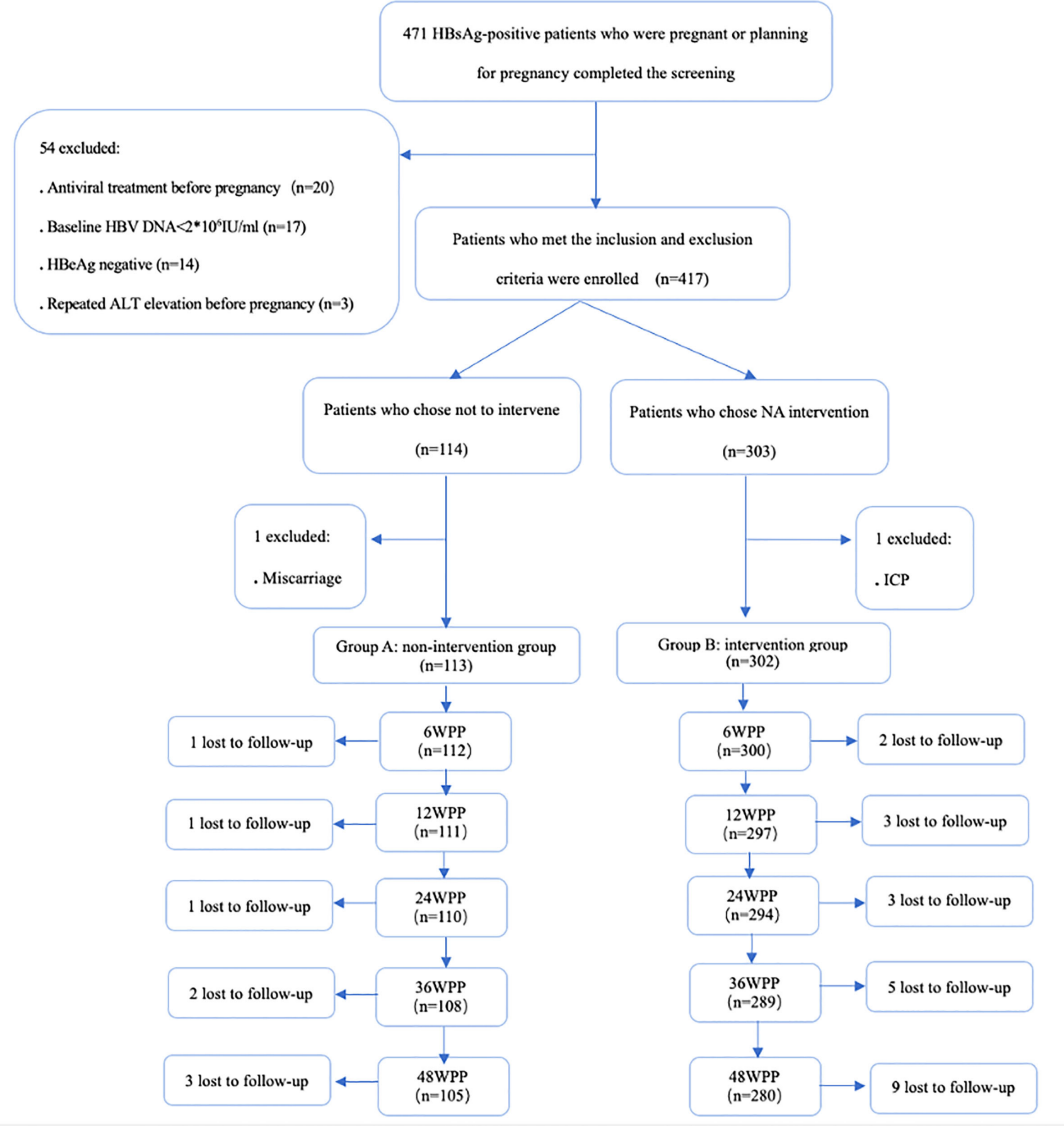
Flow chart of enrollment and follow-up of women who were chronic HBV carriers. HBsAg hepatitis B surface antigen, HBV hepatitis B virus, HBeAg hepatitis B e antigen, ALT alanine aminotransferase, NA nucleos(t)ide analogue, ICP intrahepaticcholestasis of pregnancy, WPP weeks postpartum.

A total of 471 HBsAg-positive women who were pregnant or were planning for pregnancy were screened in this study (**Figure 1**), and 417 patients with chronic HBV infection met the inclusion criteria, 114 patients joined the non-intervention group during pregnancy (group A) and 303 patients joined the NA intervention group during pregnancy (group B). Two patients, one in group A and one in group B, were excluded during pregnancy due to unexpected causes, and the follow-up and lost follow-up of 415 pregnant women included in the analysis of pregnancy were shown in **Figure 1**.

There were no significant differences in age, the baseline HBV DNA, HBeAg, and ALT levels, and the genotypes of patients between group A and group B (**Table 1**). The median time of the initiation of the NA intervention in group B was 28 weeks of gestation.

**Table 1:** General information of the two groups.

General information^a^	Group Anon-intervention group (n=113)	Group Bintervention group (n=302)	*P-*value
Age (years)	27.1 ± 4.3	28.9 ± 4.2	0.400
Baseline HBV DNA (log_10_ IU/ml)	7.6 ± 0.5	7.8 ± 0.6	0.327
Baseline HBeAg (log_10_ COI)	3.0 ± 0.2	3.1 ± 0.4	0.141
Baseline ALT (U/L)	20.1 (15.9-29.9)	26.3 (19.0-39.2)	0.050
Genotype			0.401
B	33 (29.2%)	72 (23.9%)	
C	80 (70.8%)	229 (75.8%)	
A Weeks of intervention	0 (0.0%) NA	1 (0.3%) 28 (24-28)	—
Types of intervention medications (TDF/LDT)	NA	TDF 203 (67.2%) LDT 99 (32.8%)	—

HBV hepatitis B virus, HBeAg hepatitis B e antigen, ALT alanine aminotransferase, TDF tenofovir disoproxil, LDT telbivudine, NA not available.

^a^General information were presented as mean ± standard deviation, median (interquartile range) or n (%).

### 3.2 Hepatitis flares during pregnancy

The rate of abnormal liver function during pregnancy was 20.9% (87/415), including 34 (8.2%) with mildly abnormal liver function, 53 (12.8%) with hepatitis flares, and 8 (1.9%) with hepatitis exacerbation. Among the 53 cases of hepatitis flares during pregnancy, 46 happened in non-intervention state, an incidence rate of 11.1% (46/415). Seven patients in group B had hepatitis flares during NA intervention, an incidence rate of 2.3% (7/302). The incidence rate of hepatitis flares in non-intervention state during pregnancy was significantly higher than that in intervention state, the difference was statistically significant (11.1% vs 2.3%, P < 0.001). There was no significant difference in the incidence of hepatitis flares with or without NA intervention during pregnancy, with 17.7% (20/113) and 10.9% (33/302) in group A and B, respectively (**Table 2**). However, all the 8 cases with hepatitis exacerbation occurred in non-intervention state,and all of them received NA for antiviral therapy.

**Table 2:** Incidence of hepatitis flare during pregnancy in two groups.

	Group A non-intervention group (n=113)	Group B intervention group (n=302)	*P-*value
Proportion of mildly abnormal liver function^※^ n (%)	5 (4.4%)	29 (9.6%)	0.087
Hepatitis flare* incidence n (%)	20 (17.7%)	33 (10.9%)	0.066
Proportion of hepatitis exacerbation^★^ n (%)	1 (0.9%)	7 (2.3%)	0.345
Time of hepatitis flare onset(week)^a^	14 (12-32)	17 (13-28)	0.730
Hepatitis flare incidence at each follow-up time point**^§^**			
First trimester	6/113 (5.3%)	8/302 (2.6%)	0.181
Second trimester	13/107 (12.1%)	18/294 (6.1%)	**0.046**
Third trimester	1/94 (1.1%)	7/276 (2.5%)	0.662
The level of HBV DNA at the onset of hepatitis flare (log_10_ IU/ml)^b^	7.7 ± 0.7	7.3 ± 1.1	0.570

HBV hepatitis B virus.

^a^median (interquartile range).

^b^mean ± standard deviation.

※Mildly abnormal liver function, upper limit of normal (ULN) < alanine aminotransferase(ALT)< 2 ×ULN (ULN was 40 U/L).

*Hepatitis flare, ALT ≥ 2 ×ULN.

★Hepatitis exacerbation, ALT ≥ 10 × ULN or Total bilirubin ≥ 5 × ULN, ULN was 17.1umol/L.

Patients with abnormal liver function test results were re-examined within 2 weeks, and any liver injury-inducing factors, such as alcohol, fat, immunity, and drugs, had to be excluded by a clinicians’ assessment.

§ When calculating the incidence rate of hepatitis flare at each follow-up time point during pregnancy, the total number of patients at each follow-up time point did not include patients who have had hepatitis flares before and those who were lost to follow-up.

P<0.05 is shown in bold.

In terms of the time of hepatitis flares, the second trimester was more common, with 65.0% (13/20) of patients with hepatitis flares during pregnancy in group A experienced hepatitis flares and 54.5% (18/33) of patients with hepatitis flares during pregnancy in group B experienced hepatitis flares in the second trimester.

### 3.3 Hepatitis flares during postpartum

#### 3.3.1 Incidence of postpartum hepatitis flares

After 48 weeks of postpartum follow-up, 30 patients were lost to follow-up. The 53 patients with hepatitis flares during pregnancy were not included in the analysis of postpartum hepatitis flares. Therefore, 332 patients (85 in group A and 247 in group B) were included for the overall postpartum analysis, including 132 (39.7%) with mild abnormal liver function, 148 (44.6%) with hepatitis flares. The incidence rate of hepatitis flares during the postpartum period was significantly higher than that during pregnancy (44.6% vs 12.8%, P < 0.001, **Table 3**). Further comparison of the incidence rates of hepatitis flares in each group during pregnancy and the postpartum period also showed that the incidence rate of hepatitis flares during the postpartum period was significantly higher than that during pregnancy (group A: 41.2% vs 17.7%, P < 0.001; group B: 45.7% vs 10.9%, P < 0.001; **Table 3**). The incidence rate of hepatitis exacerbation in the postpartum period was 4.8% (16/332), which was also higher than the 1.9% (8/415) during pregnancy (P = 0.026, **Table 3**). Comparing the incidence rates of hepatitis exacerbation between the two groups, group A was slightly higher than group B, which were 9.4% (8/85) and 3.2% (8/280), respectively, P=0.046.

**Table 3:** Comparison of incidence of hepatitis flare during pregnancy and postpartum.

	Total			Group A non-intervention group			Group B intervention group		
	Pregnancy (n=415)	Postpartum (n=332)	*P-*value	Pregnancy (n=113)	Postpartum (n=85)	*P-*value	Pregnancy (n=302)	Postpartum (n=247)	*P-*value
Hepatitis flare* incidence^a^	53 (12.8%)	148 (44.6%)	**<0.001**	20 (17.7%)	35 (41.2%)	**<0.001**	33 (10.9%)	113 (45.7%)	**<0.001**
Hepatitis exacerbation★ incidence^a^	8 (1.9%)	16 (4.8%)	**0.026**	1 (0.9%)	8 (9.4%)	**0.012**	7 (2.3%)	8 (3.2%)	0.510

^a^n (%).

*Hepatitis flare, ALT ≥ 2 × ULN.

★Hepatitis exacerbation, ALT ≥ 10 × ULN or Total bilirubin ≥ 5 × ULN, ULN was 17.1umol/L.

Patients with abnormal liver function test results were re-examined within 2 weeks, and any liver injury-inducing factors, such as alcohol, fat, immunity, and drugs, had to be excluded by a clinicians’ assessment.

P<0.05 is shown in bold.

In group B, about 6 weeks postpartum, 129 patients stopped NA, and the incidence of hepatitis flares in these patients was 34.1% (44/129). The other 138 patients did not stop NA and the incidence of hepatitis flares in these patients was 33.3% (46/138).The difference was not significant (P=0.893).

#### 3.3.2 Time of postpartum hepatitis flares

**Figure 2:**
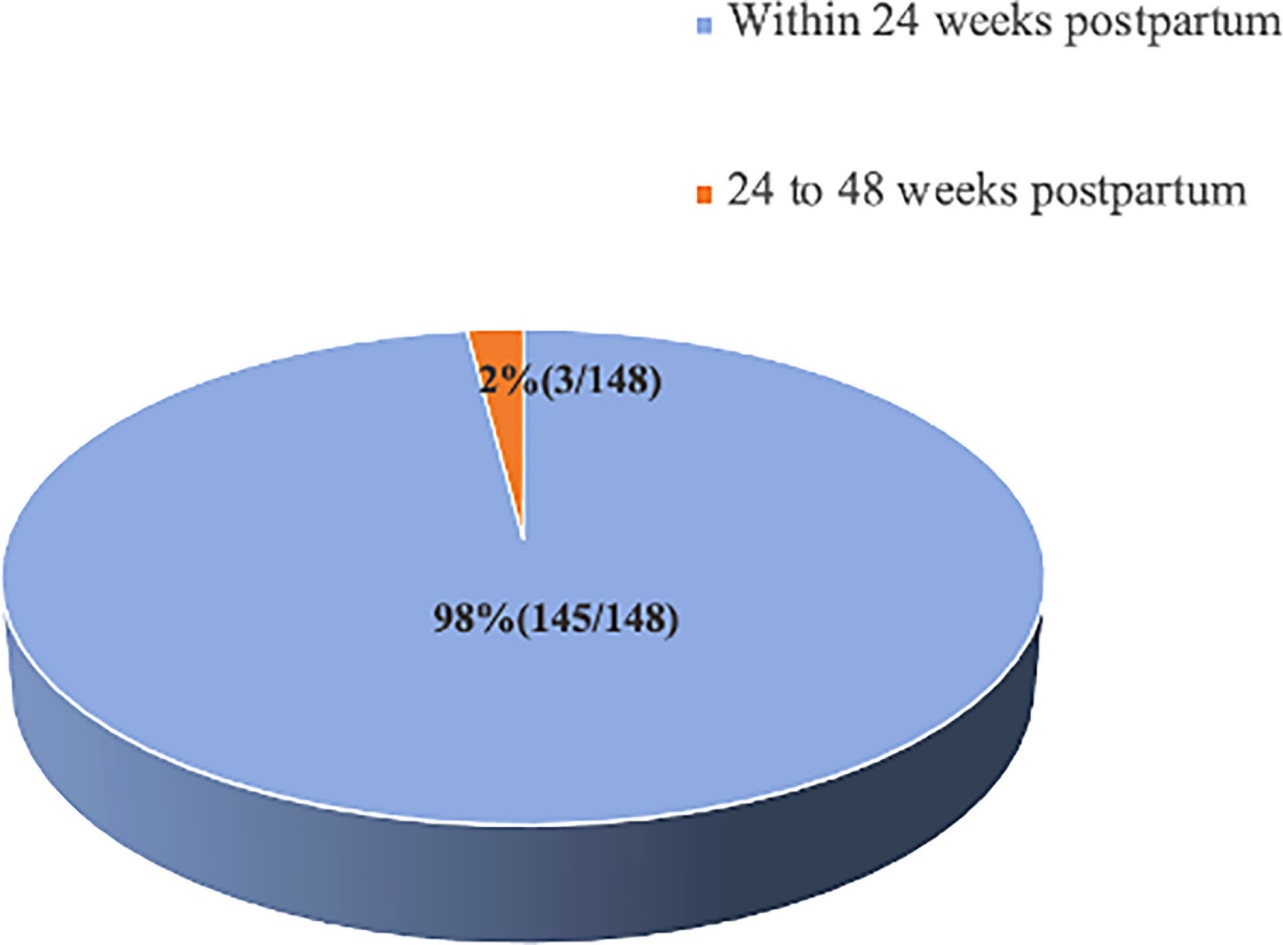
Distribution of postpartum hepatitis flare.

The median time of onset of postpartum hepatitis flares in group A and group B was both about 6 weeks postpartum, and the onset of hepatitis flares peaked at approximately 6 weeks postpartum (**Table 4**). A total of 148 patients had postpartum hepatitis flares, 81.8% (121/148) of them occurring approximately 6 weeks postpartum. The incidence rate of hepatitis flares at this time point was significantly higher than that at other follow-up time points (**Table 4**). Within 24 weeks postpartum, the cumulative number of hepatitis flare cases was 145, accounting for 98.0% of the total number of cases with hepatitis flares during the entire postpartum follow-up period. Only three cases of hepatitis flares were between the 24^th^ week and the 48^th^ week of the 48-week follow-up period, accounting for 2.0% of all postpartum hepatitis flare cases (**Figure 2**).

**Table 4:** Incidence of postpartum hepatitis flare in the two groups.

	Group A non-intervention group	Group B intervention group	*P-*value
Proportion of mildly abnormal liver function^※^	31/85 (36.5%)	101/247 (40.9%)	0.473
Hepatitis flare* incidence	35/85 (41.2%)	113/247 (45.7%)	0.464
Proportion of hepatitis exacerbation^★^	8/85 (9.4%)	8/247 (3.2%)	**0.046**
Time of hepatitis flare onset (week)^a^	6 (4-7)	6 (5-8)	0.257
Hepatitis flare incidence at each follow-up time point**^§^**			
6 ± 2WPP	31/92 (33.7%)	90/267 (33.7%)	0.998
12 ± 2WPP	2/60 (3.3%)	14/174 (8.0%)	0.342
24 ± 2WPP	1/57 (1.8%)	7/157 (4.5%)	0.607
36 ± 2WPP	0/54 (0.0%)	0/145 (0.0%)	—
48WPP	1/51 (2.0%)	2/136 (1.5%)	1.000
The level of HBV DNA at the onset of hepatitis flare (log_10_ IU/ml)^a^	7.6 (7.1-8.2)	4.4 (3.3-6.6)	**<0.001**

HBV hepatitis B virus, WPP weeks postpartum.

^a^median (interquartile range).

※Mildly abnormal liver function, upper limit of normal (ULN) < alanine aminotransferase (ALT)< 2 ×ULN (ULN was 40 U/L).

*Hepatitis flare, ALT ≥ 2 ×ULN.

★Hepatitis exacerbation, ALT ≥ 10 × ULN or Total bilirubin ≥ 5 × ULN, ULN was 17.1umol/L.

Patients with abnormal liver function test results were re-examined within 2 weeks, and any liver injury-inducing factors, such as alcohol, fat, immunity, and drugs, had to be excluded by a clinicians’ assessment.

§ When calculating the incidence rate of hepatitis flare at each postpartum follow-up time point, the total number of patients at each follow-up time point did not include patients who have had hepatitis flares before and those who were lost to follow-up.

P<0.05 is shown in bold.

#### 3.3.3 Virological parameters during postpartum hepatitis flares

Within the 48 weeks of the postpartum follow-up, the cumulative number of patients with postpartum hepatitis flares in non-intervention group (group A) was 35, and the median value of HBV DNA during hepatitis flares was 7.6 log_10_ IU/ml; the cumulative number of cases with postpartum hepatitis flares in intervention group (group B) was 113, including patients with drug cessation and patients without drug cessation, and the median value of HBV DNA during hepatitis flares was 4.4 log_10_ IU/ml, significantly lower than that in group A (P < 0.001). In group B, the HBV DNA levels at the onset of hepatitis flares were compared between patients with drug cessation and patients without drug cessation. The former had 67 cases of hepatitis flares and the median value of HBV DNA was higher, 5.6 log_10_ IU/ml; the latter had 46 cases of hepatitis flares and the median value of HBV DNA was lower, 3.3 log_10_ IU/ml, the difference was significant (P < 0.001).

## 4 Discussion

According to the recommendations of the recent guidelines for the management of hepatitis B (7–10), NAs are widely used in pregnant chronic HBV carriers in China to prevent maternal-to-child transmission. We conducted a prospective cohort study to investigate whether drug cessation after NA intervention was safe for mothers and the characteristics of the hepatitis flares in pregnant and postpartum women. The results of our study suggested that the overall incidence rates of hepatitis flares and hepatitis exacerbation were 12.8% and 1.9% in 415 chronic HBV carriers during pregnancy. These results were consistent with previous studies. The retrospective study by Kushner (11) suggested that the incidence of hepatitis flares during pregnancy in women with chronic HBV infection was 14% and that the incidence rate of ALT ≥ 10 × ULN was 2%. Our study also observed that the incidence rate of hepatitis flares during pregnancy was 11.1% (46/415) in non-intervention state, which was much higher than that (2.3%, 7/302) in intervention state. This suggests that hepatitis flares during pregnancy might not be associated with NA intervention because hepatitis flares occurred regardless of whether the intervention was applied, and the incidence of flares in non-intervention state was higher.The incidence of hepatitis flares might be mainly due to changes in immune function caused by physiological changes during pregnancy. However, there were inconsistent reports. Bzowej et al. (12) reported that the incidence rate of hepatitis flares in pregnant women with chronic HBV infection without intervention was 3.4% (5/149), which was comparable to the incidence rate of flares in pregnant women with intervention (2.0%).

In this study, among the 332 patients who were followed up to 48 weeks postpartum and included in the postpartum analysis, the incidence rate was 39.7% for mildly abnormal liver function, 44.6% for hepatitis flares, and 4.8% for hepatitis exacerbation. In the non-intervention group and the intervention group, mildly abnormal liver function and hepatitis flares were 36.5% and 40.9%, 41.2% and 45.7%, respectively, and the differences were not significant. But the proportion of hepatitis exacerbation in the non-intervention group (9.4%) was higher than that in the intervention group (3.2%), the difference was significant. Pan et al. (5) reported that in pregnant women with a high viral load of HBV, the incidence rates of abnormal ALT (ALT > 40 U/L) and severe hepatitis (ALT > 400 U/L) between 5 and 28 weeks postpartum were 45% and 1% in the TDF intervention group and 30% and 3% in the non-intervention group respectively. The incidence rates of mildly abnormal liver function in the study of Pan et al. were consistent with the results of our study, but there was no difference in the proportion of severe hepatitis in the TDF intervention group and the non-intervention group, which was inconsistent with the results of our study. In this study, we specifically subgrouped the proportion of hepatitis flares (ALT ≥ 2 × ULN), which can reach 41.2% in the non-intervention group and 45.7% in the intervention group respectively during postpartum period. According to the domestic and foreign hepatitis B guidelines (7–9) recommended standards for treatment, people with hepatitis flares are the focus of the recommended population in need of treatment, especially worthy of clinical attention.

Besides, our study also suggested that regardless of whether NA intervention was applied, the incidence rates of hepatitis flares and hepatitis exacerbation was significantly higher in the postpartum period than during pregnancy (44.6% vs 12.8%, P < 0.001; 4.8% vs 1.9%, P = 0.026). Hepatitis flares could not be avoided even during NA intervention, and the incidence rates of hepatitis flares were 2.3% in pregnant women during NA intervention and 33.3% in postpartum women. Bzowej (12) et al. also had similar report that the incidence of hepatitis flares (ALT > 100U/L) during NA intervention in pregnant women with chronic HBV infection was 2%. The incidence rate of abnormal ALT (ALT > 40 U/L) was 34% during NA intervention within 12 weeks postpartum reported by Liu (13). The results of these studies all suggested that whether the pregnant or postpartum women were in the state of intervention, drug cessation or non-intervention, the changes in ALT level were needed to be observed closely, especially the postpartum women required closer follow-up.

Regarding the time of onset of hepatitis flares during pregnancy, it had been reported (13) that about half of hepatitis flares occurred in the first and second trimester. The findings of our study showed the median time of onset of hepatitis flares was 14 weeks and 17 weeks of gestation in group A and group B, respectively and the hepatitis flares occurring in the second trimester in group A and group B accounted for 65.0% and 54.5% of all flare cases in the respective groups during pregnancy. In addition, our study also observed that the incidence of postpartum flares peaked at approximately 6 weeks postpartum, and the incidence rates of flares at this time point in group A and group B (33.7% and 33.7%) were significantly higher than those at other follow-up time points. However, some studies reported that the peak of postpartum hepatitis flares onset was about 12 weeks postpartum (14, 15). The differences may be associated with the differences in the time of the first postpartum visit and the interval between follow-up visits.

Regarding the safe time for drug cessation, 92.3% (385/417) of patients completed the 48-week follow-up, and 98% of postpartum hepatitis flares (145/148) occurred within 24 weeks of postpartum, only 2% after 24 weeks. A retrospective study (16) showed that 96% of abnormal liver function occurred within 6 weeks postpartum in postpartum women who were chronic HBV carriers followed up to 6 months postpartum. Another article (17) reported that 93.3% (42/45) of abnormal liver function occurred within 3 months postpartum in a sample of 114 pregnant women with chronic HBV infection that was followed up to 6 months postpartum. The findings of these two studies above were different from the results of our study. According to our data, although the incidence rate of postpartum hepatitis flares was already high within 12 weeks postpartum, eight new cases of hepatitis flares and one case of hepatitis exacerbation occurred from 12 weeks to 24 weeks postpartum, and only 3 new hepatitis flares occurred from 24 weeks to 48 weeks. Therefore, we believe that postpartum follow-up should be conducted for at least 24 weeks, which can cover 98% of postpartum hepatitis flare cases.

In addition, the HBV DNA load varied significantly among the patients with hepatitis flares in the intervention group in this study. Virological rebound occurred first in patients who stopped taking NA postpartum, and the HBV DNA level increased (5.6 log10 IU/ml) and then could cause hepatitis flares, which was in line with the general pattern of hepatitis flares, namely, high viral load and high ALT level. However, the hepatitis flare in patients who did not stop NA was different from the above. Even when the HBV DNA load was lower (3.3 log10 IU/ml), the ALT level was still elevated. The pattern of hepatitis flares in patients who did not stop NA was characterized by high ALT level but low viral load. The difference between the pattern of hepatitis flare before drug cessation and that after drug cessation was related to the different status of drug administration. In general, when the HBV DNA load is low, it usually does not trigger hepatitis flares. If the ALT level increases when the viral load declines, it is more likely to indicate the enhancement of immune function and the transition from immune tolerance to immune clearance period. In addition, other liver injury–inducing factors, such as fatty liver and drug-induced hepatitis, were excluded in this study. Pregnancy was generally considered to be in a relatively immunosuppressive state (18) to avoid rejection, which is conducive to the growth and development of the offspring, but the immune function is relatively enhanced during the postpartum period (2, 19). In addition, with high viral load during pregnancy, the body tended to be immune tolerant, and the decline of viral load after delivery is equivalent to weakened immune tolerance. Some scholars also have reported that many autoimmune diseases can be relieved during pregnancy but often recur after delivery (20). These mechnisms might partly explain the reason why the incidence of postpartum flares (44.6%) was significantly higher than that (12.8%) of during pregnancy. However,it has not been reported whether the high ALT level but low viral load hepatitis flares observed in this study has a similar immune mechanism. To explore the therapeutic effects under this condition, our team (21) treated patients with pegylated interferon and NA for 96 weeks and achieved satisfactory treatment outcomes: The HBeAg clearance rate can reach 56.7% (17/30), and the HBsAg clearance rate was 26.7% (8/30), suggesting that women who did not stop treatment after delivery may achieve better therapeutic effects if they had antiviral therapy timely for postpartum hepatitis flares.

In summary, the status of immune tolerance in pregnant women who were chronic HBV carriers was not always unchanged. Regardless of whether NA intervention was applied, a certain proportion of hepatitis flares occurred during pregnancy and postpartum period, and the incidence rate of hepatitis flares during postpartum(44.6%) was significantly higher than that during pregnancy (12.8%). The peak of postpartum hepatitis flare was in 6 weeks. Since 98% of the postpartum hepatitis flares occurred within 24 weeks postpartum, patients should be followed up for at least 24 weeks postpartum to determine whether they can safely stop the treatment.

## Data availability statement

The raw data supporting the conclusions of this article will be made available by the authors, without undue reservation.

## Ethics statement

The studies involving human participants were reviewed and approved by The ethics committee of Beijing Youan Hospital, Capital Medical University. The patients/participants provided their written informed consent to participate in this study. Written informed consent was obtained from the individual(s) for the publication of any potentially identifiable images or data included in this article.

## Author contributions

XC, XW, and AS conceived and designed the protocol and study. AS, XL and SR collected the data. XW analyzed the data and drafted the manuscript. YZ performed the follow up of patients. JL, SZ, and LM guided statistical analysis. XC contributed to the interpretation of the results and critical revision of the manuscript for important intellectual content and approved the final version of the manuscript. All authors contributed to the article and approved the submitted version.

## Funding

This work was supported by the Capital Clinical Diagnostic Techniques and the Translational Application Projects (Z211100002921059), Chinese National Natural Science Foundation (81900537), Beijing Hospitals Authority Clinical medicine Development of special funding support (XMLX202125), and the Capital Health Research and Development Projects (2020–1–2181).

## Acknowledgments

The authors thank Yunxia Zhu, the director of Obstetrics and Gynecology, Beijing Youan Hospital, Capital Medical University, for supporting the enrollment and follow-up of patients.

## Conflict of interest

The authors declare that the research was conducted in the absence of any commercial or financial relationships that could be construed as a potential conflict of interest.

The handling editor declared a shared parent affiliation with the authors at the time of review.

## Publisher’s note

All claims expressed in this article are solely those of the authors and do not necessarily represent those of their affiliated organizations, or those of the publisher, the editors and the reviewers. Any product that may be evaluated in this article, or claim that may be made by its manufacturer, is not guaranteed or endorsed by the publisher.

## Abbreviations

HBV, hepatitis B virus; HBsAg, hepatitis B surface antigen; NA, nucleos(t)ide analogue; HBeAg, hepatitis B e antigen; ALT, alanine aminotransferase; ULN, upper limit of normal; TDF tenofovir disoproxil, LDT telbivudine.

## References

[1] Cui F Luo H Wang F Zheng H Gong X Chen Y. Evaluation of policies and practices to prevent mother to child transmission of hepatitis b virus in China: results from China GAVI project final evaluation. Vaccine (2013) 31 Suppl 9:J36–42. doi: 10.1016/j.vaccine.2012.11.061 24331019

[2] Xu WM Cui YT Wang L Yang H Liang ZQ Li XM. Lamivudine in late pregnancy to prevent perinatal transmission of hepatitis b virus infection: A multicentre, randomized, double-blind, placebo-controlled study. J Viral Hepat. (2009) 16:94–103. doi: 10.1111/j.1365-2893.2008.01056.x 19175878

[3] Zou H Chen Y Duan Z Zhang H Pan C. Virologic factors associated with failure to passive-active immunoprophylaxis in infants born to HBsAg-positive mothers. J Viral Hepat. (2012) 19:e18-25. doi: 10.1111/j.1365-2893.2011.01492.x 22239517

[4] Han GR Cao MK Zhao W Jiang HX Wang CM Bai SF. A prospective and open-label study for the efficacy and safety of telbivudine in pregnancy for the prevention of perinatal transmission of hepatitis b virus infection. J Hepatol (2011) 55:1215–21. doi: 10.1016/j.jhep.2011.02.032 21703206

[5] Pan CQ Duan Z Dai E Zhang S Han G Wang Y. Tenofovir to prevent hepatitis b transmission in mothers with high viral load. N Engl J Med (2016) 374:2324–34. doi: 10.1056/NEJMoa1508660 27305192

[6] Zhang H Pan CQ Pang Q Tian R Yan M Liu X. Telbivudine or lamivudine use in late pregnancy safely reduces perinatal transmission of hepatitis b virus in real-life practice. Hepatology (2014) 60 :468-76. doi: 10.1002/hep.27034 25187919 PMC4282428

[7] Chinese Society of Hepatology CMA Chinese Society of Infectious Diseases CMA Hou JL Lai W. [The guideline of prevention and treatment for chronic hepatitis b: A 2015 update]. (2015) 23:888–905. Zhonghua Gan Zang Bing Za Zhi. doi: 10.3760/cma.j.issn.1007-3418.2015.12.034 PMC12677373 26739464

[8] Terrault NA Bzowej NH Chang KM Hwang JP Jonas MM Murad MH. American Association for the study of liver d. AASLD guidelines for treatment of chronic hepatitis b. Hepatology (2016) 63:261–83. doi: 10.1002/hep.28156 PMC5987259 26566064

[9] European Association for the Study of the Liver Electronic address eee, European association for the study of the l. EASL 2017 clinical practice guidelines on the management of hepatitis b virus infection. J Hepatol (2017) 67:370–98. doi: 10.1016/j.jhep.2017.03.021 28427875

[10] Sarin SK Kumar M Lau GK Abbas Z Chan HL Chen CJ. Asian-Pacific clinical practice guidelines on the management of hepatitis b: A 2015 update. Hepatol Int (2016) 10:1–98. doi: 10.1007/s12072-015-9675-4 PMC4722087 26563120

[11] Kushner T Shaw PA Kalra A Magaldi L Monpara P Bedi G. Incidence, determinants and outcomes of pregnancy-associated hepatitis b flares: A regional hospital-based cohort study. Liver Int (2018) 38:813–20. doi: 10.1111/liv.13594 PMC12136009 28941137

[12] Bzowej NH Tran TT Li R Belle SH Smith CI Khalili M. Total alanine aminotransferase (ALT) flares in pregnant north American women with chronic hepatitis b infection: Results from a prospective observational study. Am J Gastroenterol (2019) 114:1283–91. doi: 10.14309/ajg.0000000000000221 PMC7132838 31082876

[13] Liu J Wang J Jin D Qi C Yan T Cao F. Hepatic flare after telbivudine withdrawal and efficacy of postpartum antiviral therapy for pregnancies with chronic hepatitis b virus. J Gastroenterol Hepatol (2017) 32:177–83. doi: 10.1111/jgh.13436 27161163

[14] Chang CY Aziz N Poongkunran M Javaid A Trinh HN Lau D. Serum alanine aminotransferase and hepatitis b DNA flares in pregnant and postpartum women with chronic hepatitis b. Am J Gastroenterol (2016) 111:1410–5. doi: 10.1038/ajg.2016.296 27456990

[15] Nguyen V Tan PK Greenup AJ Glass A Davison S Samarasinghe D. Anti-viral therapy for prevention of perinatal HBV transmission: extending therapy beyond birth does not protect against post-partum flare. Aliment Pharmacol Ther (2014) 39:1225–34. doi: 10.1111/apt.12726 24666381

[16] Cao Y Yi W Liu M Cai H. Postpartum observation of changes in liver function of pregnant women with hepatitis b virus infection in immune tolerant phase. Chin J Exp Clin Infect Dis (Electronic. Edition) (2013) 7:111–4. doi: 10.3877/cma.j.issn.1674-1358.2013.03.029

[17] An X Han G Jiang H Wang C. Observation on postpartum changes in liver function of pregnant women with chronic HBV infection. Occup Health (2016) 32:498–501. doi: 10.13329/j.cnki.zyyjk.2016.0114

[18] Schjenken J Tolosa J Paul J Clifton V Smith R. Mechanisms of maternal immune tolerance during pregnancy. Rijeka, Croatia: INTECH. (2012).

[19] Aluvihare VR Kallikourdis M Betz AG. Regulatory T cells mediate maternal tolerance to the fetus. Nat Immunol (2004) 5:266–71. doi: 10.1038/ni1037 14758358

[20] Warning JC McCracken SA Morris JM. A balancing act: mechanisms by which the fetus avoids rejection by the maternal immune system. Reproduction (2011) 141:715–24. doi: 10.1530/REP-10-0360 21389077

[21] Lu J Zhang S Liu Y Du X Ren S Zhang H. Effect of peg-interferon alpha-2a combined with adefovir in HBV postpartum women with normal levels of ALT and high levels of HBV DNA. Liver Int (2015) 35:1692–9. doi: 10.1111/liv.12753 25438657

