# Supplementary figures and images for: Clinical characteristics of hepatitis flares during pregnancy and postpartum in Chinese chronic hepatitis B virus carriers—a prospective cohort study of 417 cases
Source: Front Immunol. 2022 Oct 13;13:1031291. doi: 10.3389/fimmu.2022.1031291 (PMC9606458; doi:10.3389/fimmu.2022.1031291)

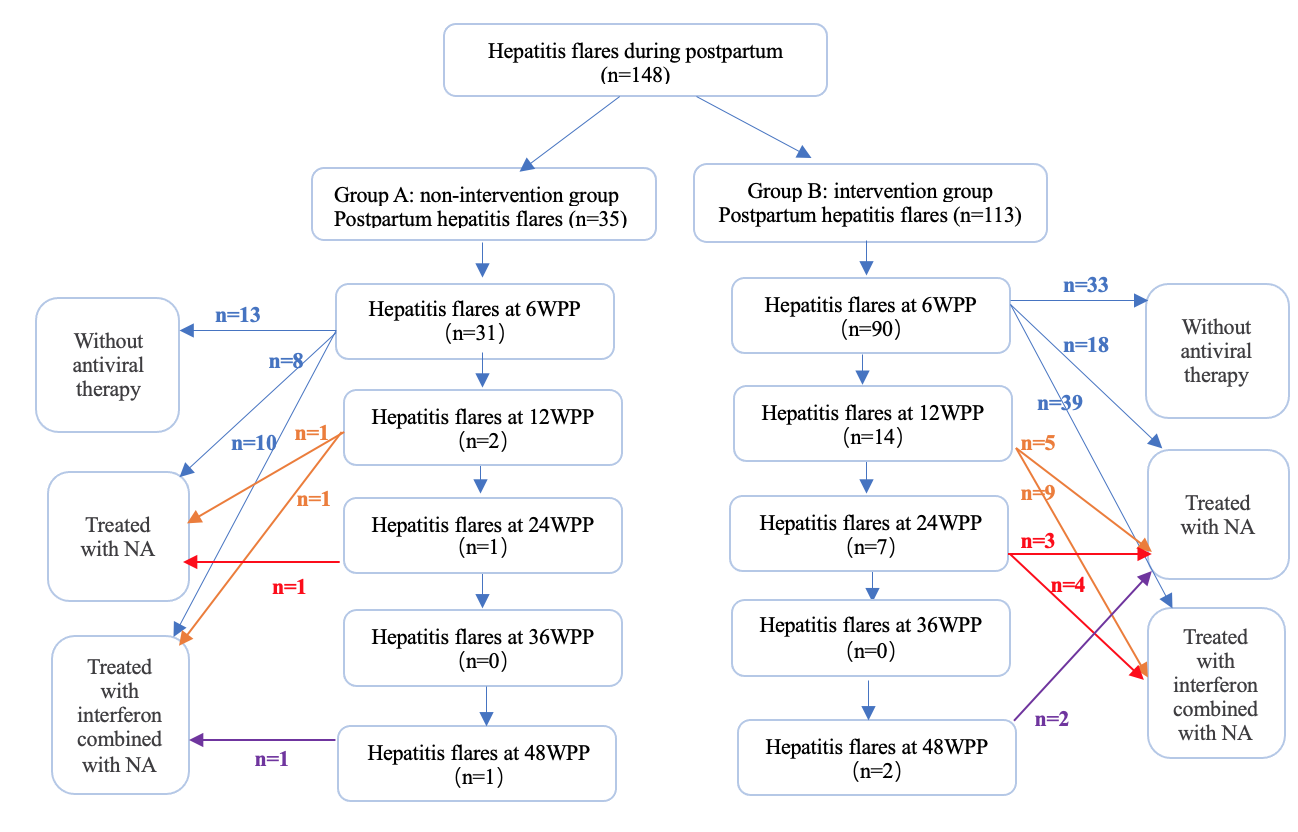


Figure S1.Treatment course with all the patient numbers and hepatitis flares for each group

Supplement: Supplementary file 1 [file DataSheet_1.docx]
